# Clinical Performance of GalaFLEX® (Poly-4-Hydroxybutyrate) Scaffold Application in Aesthetic and Reconstructive Breast Surgery: A Retrospective Study

**DOI:** 10.1007/s00266-026-06034-4

**Published:** 2026-06-16

**Authors:** Leonardo Adolfo Spuras Stella, Bruna Lee Damasceno, Giovanna Valente Costa, Nicole Martins Stella, Vanessa Borges Valente Stella, Rosa Andréa Nogueira Laiso, Rose Eli Grassi Rici, Durvanei Augusto Maria, Gustavo Henrique Doná Rodrigues Almeida

**Affiliations:** 1 Graduate Program in Medical Sciences, School of Medicine, University of São Paulo, São Paulo, Brazil; 2 Clínica Stella, Private Practice, Americana, São Paulo, Brazil; 3 Pontifical Catholic University of Campinas, São Paulo, Brazil; 4 Graduate Program in Anatomy of Domestic and Wild Animals, School of Veterinary Medicine and Animal Science, University of São Paulo, São Paulo, Brazil; 5 Laboratory of Development and Innovation, Butantan Institute, São Paulo, Brazil

**Keywords:** Poly-4-hydroxybutyrate, Breast surgery, Synthetic mesh, Biomaterial

## Abstract

**Background:**

Soft-tissue support remains a critical factor in both reconstructive and aesthetic breast surgery. Bioresorbable synthetic scaffolds, such as poly-4-hydroxybutyrate (P4HB), have been introduced as an alternative to acellular dermal matrices to provide structural reinforcement while gradually integrating into host tissue. This study evaluates the clinical indications, surgical applications, and early postoperative outcomes associated with the use of a bioresorbable P4HB scaffold in implant-based breast surgery.

**Methods:**

A retrospective review was conducted of patients who underwent breast surgery with P4HB scaffold reinforcement. Surgical indications were analyzed per treated breast and categorized as oncologic or non-oncologic. Associated procedures, including fat grafting and implant exchange versus primary implantation, were recorded when available. Postoperative complications were assessed per patient. Descriptive statistics were used to summarize surgical indications, technical approaches, and outcomes.

**Results:**

A total of 31 patients and 52 treated breasts were included. Surgical indications were evenly distributed between breasts with and without disease. All breasts with disease were managed in the context of post-mastectomy reconstruction. In breasts without disease, scaffold reinforcement was primarily used for contralateral symmetrization, followed by primary or revisional aesthetic procedures. Fat grafting was frequently performed as an adjunct technique. Most cases involved primary implant placement, with a small proportion representing revision surgery. The overall postoperative complication rate was low, with the majority of patients experiencing an uneventful recovery.

**Conclusion:**

P4HB scaffold reinforcement demonstrated a favorable safety profile and versatile clinical applicability in both reconstructive and aesthetic breast surgery. Its integration into implant-based procedures, often in combination with adjunctive techniques, supports its role as a reliable option for soft-tissue support. Further studies with longer follow-up and comparative designs are needed to better define long-term outcomes and optimize indications.

**Level of Evidence IV:**

This journal requires that authors assign a level of evidence to each article. For a full description of these Evidence-Based Medicine ratings, please refer to the Table of Contents or the online Instructions to Authors www.springer.com/00266.

## Introduction

Breast cancer remains one of the most prevalent malignancies affecting women worldwide and continues to represent a major cause of cancer-related mortality [1, 2]. Despite advances in screening strategies and oncologic treatments, a substantial proportion of patients still requires mastectomy as part of disease control or risk reduction [3]. As survival rates increase, attention has progressively shifted toward long-term outcomes and quality of life, highlighting the importance of breast reconstruction as an integral component of comprehensive breast cancer care [4]. Breast reconstruction extends beyond the restoration of breast volume, aiming to reestablish contour, symmetry, and projection, thereby contributing to physical rehabilitation and psychosocial well-being [4]. Implant-based reconstruction remains one of the most commonly adopted approaches; however, it is frequently associated with challenges related to insufficient soft-tissue support, particularly in patients with thin skin flaps, prior radiotherapy, or extensive resections [5].

Loss of structural support of the lower pole and inframammary fold is a well-recognized cause of complications such as inferior implant displacement, contour deformities, recurrent ptosis, and asymmetry [6, 7]. Traditional techniques relying solely on sutures or muscle manipulation may be inadequate in these scenarios, leading to reported reoperation rates ranging from 10% to 30% in complex implant-based reconstructions [8]. These limitations have driven the incorporation of supportive matrices as adjuncts to improve implant stability and aesthetic predictability [9]. Biological and synthetic matrices have been widely introduced in breast reconstruction with the aim of reinforcing the soft-tissue envelope. However, their use is not exempt from complications [10]. Studies evaluating acellular dermal matrices have reported complication rates ranging from 15 to 35%, with seroma formation occurring in up to 20% of cases, surgical site infection in 5–15%, and skin flap necrosis in up to 10%, particularly in irradiated patients [11, 12]. Capsular contracture rates were also reported with biological matrices [13].

Synthetic non-absorbable meshes, while providing effective mechanical support, have also been associated with relevant adverse events [14]. Reported complications include chronic inflammation, persistent seroma, mesh exposure, and infection, with overall complication rates reaching 20–30% in some series [15]. Additionally, the permanent presence of synthetic material may complicate revision surgeries and limit long-term tissue adaptability [16]. Absorbable synthetic matrices have emerged as an alternative designed to mitigate these limitations by providing temporary mechanical reinforcement during the critical phases of healing, followed by gradual degradation as host tissue remodeling occurs [17]. Clinical studies evaluating absorbable meshes in breast surgery have reported lower long-term foreign body-related complications, with infection rates generally below 5–8% and reduced incidence of chronic inflammatory reactions when compared to non-absorbable materials [18, 19]. These characteristics are particularly advantageous in patients at higher risk for wound complications or those requiring revision procedures [17].

Among absorbable synthetic matrices currently available for soft-tissue reinforcement, GalaFLEX® is composed of poly-4-hydroxybutyrate (P4HB), a fully absorbable monofilament polymer that provides temporary structural support while undergoing gradual degradation over approximately 12 to 18 months [20]. The material has been primarily described in aesthetic breast procedures, particularly mastopexy and augmentation-mastopexy, where its use has been associated with stabilization of the lower pole and inframammary fold [21]. However, the current literature predominantly addresses aesthetic indications, with limited data evaluating the application of P4HB scaffolds in reconstructive breast surgery, revision procedures, or mixed clinical cohorts that integrate both aesthetic and reconstructive indications. However, comparative clinical evidence between absorbable scaffolds such as P4HB and alternative reinforcement strategies, including permanent meshes or procedures without mesh support, remains limited. Therefore, the present study was designed to describe clinical use patterns and early outcomes rather than to establish comparative effectiveness or superiority. In this context, we evaluated the clinical outcomes and safety profile associated with GalaFLEX use across a spectrum of aesthetic and reconstructive breast procedures, with particular attention to implant-based surgeries, postoperative complications, and implant stability in routine clinical practice.

## Materials and Methods

### Study Design

**Fig. 1:**
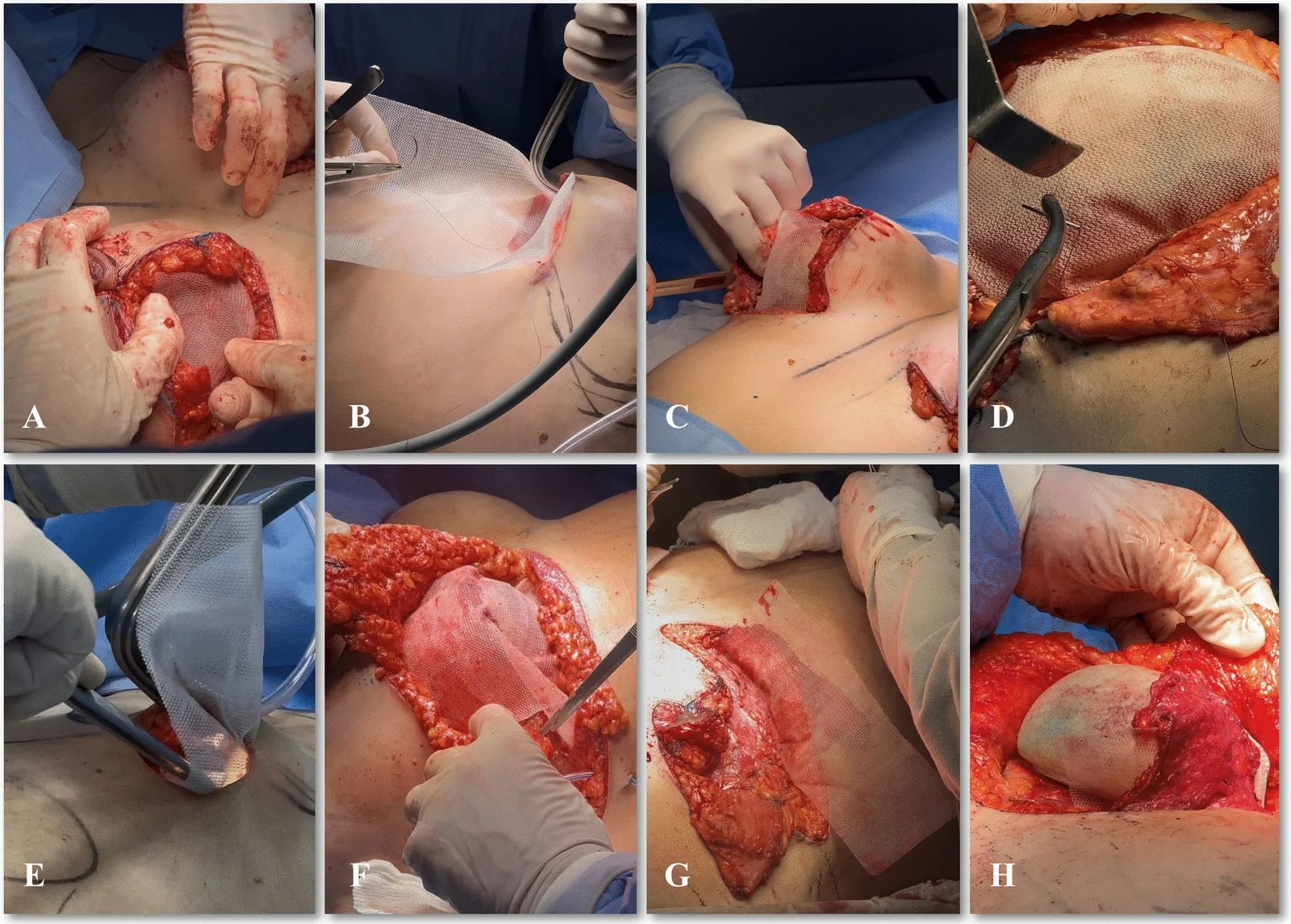
Step-by-step illustration of GalaFLEX® (poly-4-hydroxybutyrate) scaffold placement in aesthetic and reconstructive breast surgery. Preparation of the surgical pocket after tissue dissection and definition of the defect area (**A**). Trimming and shaping of the scaffold according to the dimensions and contour of the defect (**B**). Initial positioning of the scaffold within the surgical pocket, ensuring adequate coverage of the target area (**C**). Primary fixation of the scaffold to the surrounding tissues using interrupted sutures, allowing initial stabilization (**D**). Peripheral fixation and fine adjustment of the scaffold margins to optimize adaptation to the host tissues and pocket geometry (**E**). Scaffold fully positioned and stabilized within the defect, demonstrating adequate conformity and support (**F**). Redraping of the overlying soft tissues over the scaffold prior to final closure (**G**). Immediate intraoperative appearance after reconstruction, highlighting the supported breast contour and scaffold integration within the surgical site (**H**)

This study was designed as a retrospective observational case series aimed at evaluating real-world clinical applications and early postoperative outcomes associated with P4HB scaffold use, rather than performing a comparative analysis. Accordingly, no control group was included, as the primary objective was to assess feasibility, safety, and versatility across different surgical indications. The study comprised a retrospective analysis of women who underwent breast reconstruction or aesthetic mastopexy with implant placement reinforced by the absorbable synthetic mesh GalaFLEX (poly-4-hydroxybutyrate, P4HB). This bioresorbable monofilament scaffold is designed to provide temporary soft-tissue support, with progressive degradation and resorption typically occurring over 12 to 18 months [22] (Fig. 1). All procedures were performed between April and December 2025 at three hospitals in Americana, São Paulo, Brazil. Data were collected through retrospective review of medical records, including demographic characteristics, surgical variables, and postoperative outcomes. As the study relied exclusively on secondary data, with no direct patient interaction or deviation from standard clinical practice, the Institutional Ethics Committee was notified and determined that formal ethical approval was not required. Written informed consent for the publication of identifiable clinical images was obtained from all patients included in this manuscript.

### Surgical Procedures

All surgical procedures were conducted under general anesthesia. Perioperative antibiotic prophylaxis consisted of a single intravenous dose of cefazolin (2 g) administered at anesthetic induction, in combination with 1 g of intravenous tranexamic acid. Antisepsis of the surgical field was performed in two sequential steps using a 2% chlorhexidine gluconate solution. Preexisting inframammary scars, when present, were excised as part of the surgical approach. In cases involving previous breast implants, the devices were explanted and detailed information regarding manufacturer, model, and volume was documented. Standardized clinical photographs were obtained preoperatively and postoperatively following written patient consent. Management of the periprosthetic capsule was tailored according to the anatomical location of the prior implant. For implants previously positioned in the subglandular plane, total capsulectomy was routinely performed, and the excised capsule was submitted for histopathological examination, including specific assessment for breast implant-associated anaplastic large cell lymphoma (BIA-ALCL). In cases of submuscular or dual-plane placement, capsule removal was attempted with careful preservation of adjacent structures, including the ribs, intercostal musculature, and the inferior border of the pectoralis major muscle. When complete excision was not feasible due to dense adhesions or safety concerns, representative capsular specimens were harvested for pathological analysis.

In patients undergoing conversion from a submuscular to a subglandular implant pocket, the pectoralis major muscle was reinserted to its anatomical origin using a continuous locking 3-0 polydioxanone suture. Prior to placement of the absorbable synthetic scaffold, meticulous hemostasis of the implant pocket was systematically achieved. The definitive breast implant was subsequently inserted using a protective insertion sleeve to avoid mechanical disruption of the mesh. Structural reinforcement of the inframammary fold and the lateral mammary ligament was performed with absorbable sutures to enhance inferior and lateral pole support. Layered wound closure was accomplished using absorbable sutures for the deep and subcutaneous planes, followed by intradermal closure and application of a topical skin adhesive system. Surgical drains were routinely placed in reconstructive cases, while their use in aesthetic procedures was determined on an individual intraoperative basis. In selected patients, adjunctive autologous fat grafting was performed in the subdermal plane, either focally or diffusely, to improve soft-tissue coverage and breast contour.

### Breast Reconstruction

Breast reconstruction was carried out using a staged approach. During the initial procedure, performed at the time of mastectomy, a tissue expander was inserted beneath the pectoralis major muscle. After completion of the expansion phase, a second operation was undertaken to exchange the expander for a definitive implant. This procedure was performed through an inframammary incision measuring approximately 8 cm. At the time of implant exchange, the inferior border of the pectoralis major muscle was released, and both the expander and its valve were removed. Capsular adjustments, including superior and medial capsulotomies, were performed as required to optimize pocket dimensions for the definitive prosthesis. The permanent implant was positioned in a dual-plane configuration, with muscular coverage over the superior and lateral regions and subglandular placement along the inferior pole. An absorbable synthetic mesh was then tailored to match the contour of the lower pole and secured to reinforce the lateral breast envelope while providing additional support and coverage to the inferior portion of the implant. The procedure was completed with multilayered wound closure.

### Aesthetic Mastopexy or Contralateral Reconstruction

Aesthetic mastopexy associated with implant placement was performed using either a subglandular or a dual-plane approach, selected according to patient anatomy, tissue characteristics, and surgical indication. Preoperative planning included standardized skin markings, with definition of the nipple–areola complex position based on Pitanguy’s point A and delineation of the excess skin to be excised. In both techniques, a bilateral Schwartzmann maneuver was routinely employed. Inferior glandular tissue was partially resected, followed by the elevation of a dermoglandular flap measuring approximately 0.5 cm in thickness, with an average base width of 8 cm and variable vertical extension ranging from 4 to 10 cm. After thorough inspection and control of hemostasis, reinforcement of the inframammary fold and lateral breast support was performed using absorbable sutures (Vicryl® 1.0 and 3.0).

For the subglandular approach, polyurethane-coated implants were placed directly within the prepared pocket. The absorbable synthetic scaffold (GalaFLEX®) was positioned to provide additional support to the inferior and lateral poles. Despite the known tissue integration properties of polyurethane implants, mesh reinforcement was systematically applied to enhance mechanical stability, particularly in patients presenting with limited soft-tissue thickness or diminished parenchymal support. In the dual-plane technique, a transverse incision of approximately 5 cm was made along the inferior border of the pectoralis major muscle. Partial release of the muscle allowed placement of a microtextured implant, while maintaining the inferior portion of the prosthesis in the subglandular plane. When necessary, supplementary glandular resection was performed to optimize breast volume distribution and achieve symmetry. In both surgical strategies, the nipple–areola complex was transposed using either a superior or superomedial pedicle and secured at the previously marked point A. The absorbable synthetic mesh was carefully positioned to reinforce the lateral breast envelope and inferior pole, contributing to implant stabilization. Final wound closure was performed in multiple layers using absorbable sutures (Vicryl® 4.0 and Monocryl® 4.0).

### Postoperative Care

In the immediate postoperative period, all patients were fitted with a surgical compression bra. Additional thoracic support using a strap or binder was applied when indicated according to the surgical technique and intraoperative assessment. External compression was maintained for a duration ranging from 1 to 5 weeks, based on individual postoperative evolution. Postoperative antibiotic prophylaxis consisted of oral cephalexin 500 mg administered every 6 h for a period of 7–10 days. Analgesic therapy was prescribed on an individualized basis according to patient needs. Early postoperative assessments were conducted at 24 hours, 1 week, and 3 weeks following surgery. During this phase, patients underwent supervised motor physiotherapy and lymphatic drainage twice weekly for three consecutive weeks. Follow-up data were available for a minimum of 6 months in all patients, after which patients were discharged from routine follow-up, with continued access to outpatient consultation if required.

### Data Collection

Data were retrospectively extracted from medical records after completion of the study period. Patient characterization included demographic and anthropometric variables such as age, body weight, and height, which were used to calculate body mass index (BMI). Additional data encompassed lifestyle factors, including smoking status, as well as surgical history, specifically the number of prior breast procedures and the indication for the index surgery. Information regarding adjuvant treatments administered before or after surgery, including adjuvant oncological treatments, was also collected. Postoperative outcomes were reviewed, with the occurrence of complications recorded descriptively.

### Data Analysis

All collected data were coded into numerical categories and organized using Microsoft Excel® spreadsheets. Statistical analyses were performed using GraphPad Prism, version 8.0 (GraphPad Software, San Diego, CA, USA). Data distribution was explored descriptively. Descriptive statistics were applied for data presentation, including absolute and relative frequencies (n and %) for categorical variables and mean ± standard deviation for continuous variables. Given the limited number of postoperative complications observed in the cohort (*n* = 1), inferential statistical analyses were not performed. Postoperative outcomes and comparisons between patients with and without complications were therefore presented using descriptive analysis only.

## Results

A total of 31 patients were included in the study, all of whom underwent breast reconstruction or aesthetic mastopexy with implant placement reinforced by an absorbable synthetic mesh. Polyurethane-coated implants were used in subglandular approaches, whereas microtextured implants were predominantly used in dual-plane techniques. Baseline demographic and clinical characteristics are summarized in Table 1. The mean age of the cohort was 48.9 ± 16 years, with a mean body mass index (BMI) of 25.4 ± 2.9 kg/m^2^. Most patients were classified as having normal weight (58.1%), followed by overweight (32.3%) and obesity (9.7%). No patients were underweight. None of the patients were active smokers, and alcohol consumption was reported by 25.8% of the cohort.

**Table 1:** Baseline demographic and clinical characteristics of patients undergoing breast reconstruction or aesthetic mastopexy with synthetic mesh reinforcement (*n* = 31)

Variables	All (*n* = 31)	No complications (*n* = 30)	With complications (*n* = 1)
*n* (%) or mean ± SD	*n* (%) or mean ± SD	*n* (%) or mean ± SD	
Age in years	48.9 ± 16	48.1 ± 15.5	74
Smoker (%)	0	0	0
Alcohol use (%)	25.8	26.7	0
Weight (kg)	68.8 ± 9.6	68.8 ± 9.8	69
Height (cm)	164.5 ± 7.1	164.8 ± 7.1	157
BMI (kg/m^2^)	25.4 ± 2.9	25.3 ± 2.9	28
BMI classification (%)			
Underweight	0	0	0
Normal weight	58.1	60	0
Overweight	32.3	30	100
Obesity	9.7	10	0
Associated comorbidities (%)			
Diabetes	6.5	6.7	0
Arterial hypertension	9.7	10	0
Hypothyroidism	6.5	6.7	0
Previous breast surgeries (%)			
None	35.5	36.7	0
One	38.7	36.7	100
Two	19.4	20	0
Three	6.5	6.7	0
Surgical indication			
Neoplasia and reconstruction	38.7	36.7	100
Aesthetic and reconstruction	61.3	63.3	0
Preoperative radiotherapy, yes	35.5	33.3	100
Preoperative chemotherapy, yes	35.5	36.7	0

*BMI* body mass index

Regarding associated comorbidities, diabetes mellitus was present in 6.5% of patients, arterial hypertension in 9.7%, and hypothyroidism in 6.5%. Previous breast surgery was common, with only 35.5% of patients having no prior procedures. One previous breast surgery was reported in 38.7% of cases, two in 19.4%, and three in 6.5%. Surgical indications included implant-based breast reconstruction following neoplasia in 38.7% of patients, while 61.3% underwent aesthetic procedures or contralateral reconstruction. Preoperative oncologic treatments were frequent among reconstructive cases. Preoperative radiotherapy was documented in 35.5% of patients, and preoperative chemotherapy in 35.5%. One postoperative complication was observed in the cohort. This patient was older (74 years), classified as overweight (BMI = 28 kg/m^2^), had a history of one previous breast surgery, and had undergone preoperative radiotherapy. No complications were observed among the remaining 30 patients.

**Fig. 2:**
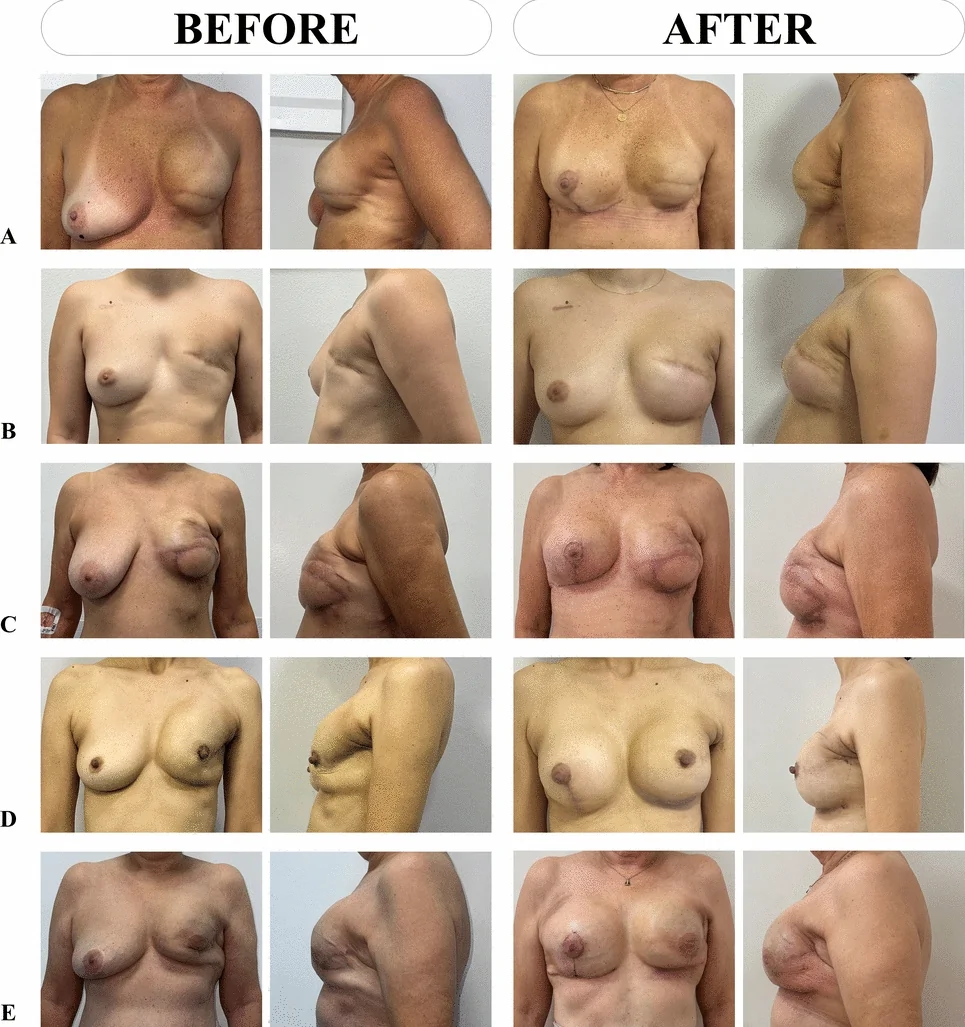
Pre- and postoperative photographs of patients undergoing breast reconstruction with GalaFLEX® (poly-4-hydroxybutyrate) mesh. In the preoperative images (left columns), post-mastectomy sequelae are evident, including breast asymmetry, volume deficiency, residual ptosis, and contour irregularities. The postoperative images (right columns) demonstrate improved breast volume, projection, and overall contour, with enhanced symmetry across the different cases (**A–E**), reflecting the contribution of the mesh to soft-tissue support and contour harmonization

Representative preoperative and postoperative clinical photographs of patients undergoing implant-based breast reconstruction are shown in Fig. 2. Preoperative images illustrate deformities commonly observed following mastectomy and prior reconstructive procedures, including volume deficiency, asymmetry, altered breast contour, and poor definition of the lower pole and inframammary fold. In several cases, lateral views demonstrate reduced anterior projection and compromised soft-tissue support, particularly along the inferior and lateral breast envelope (Fig. 2A–E, left columns). Postoperative images obtained during follow-up demonstrate restoration of breast volume and anterior projection, with improved contour of the lower pole and redefinition of the inframammary fold (Fig. 2A–E, right columns). Lateral views show stabilization of implant position and maintenance of projection without evidence of inferior displacement. These images highlight the clinical application of the absorbable synthetic scaffold in reconstructive breast surgery, while aesthetic cases are presented separately.

**Fig. 3:**
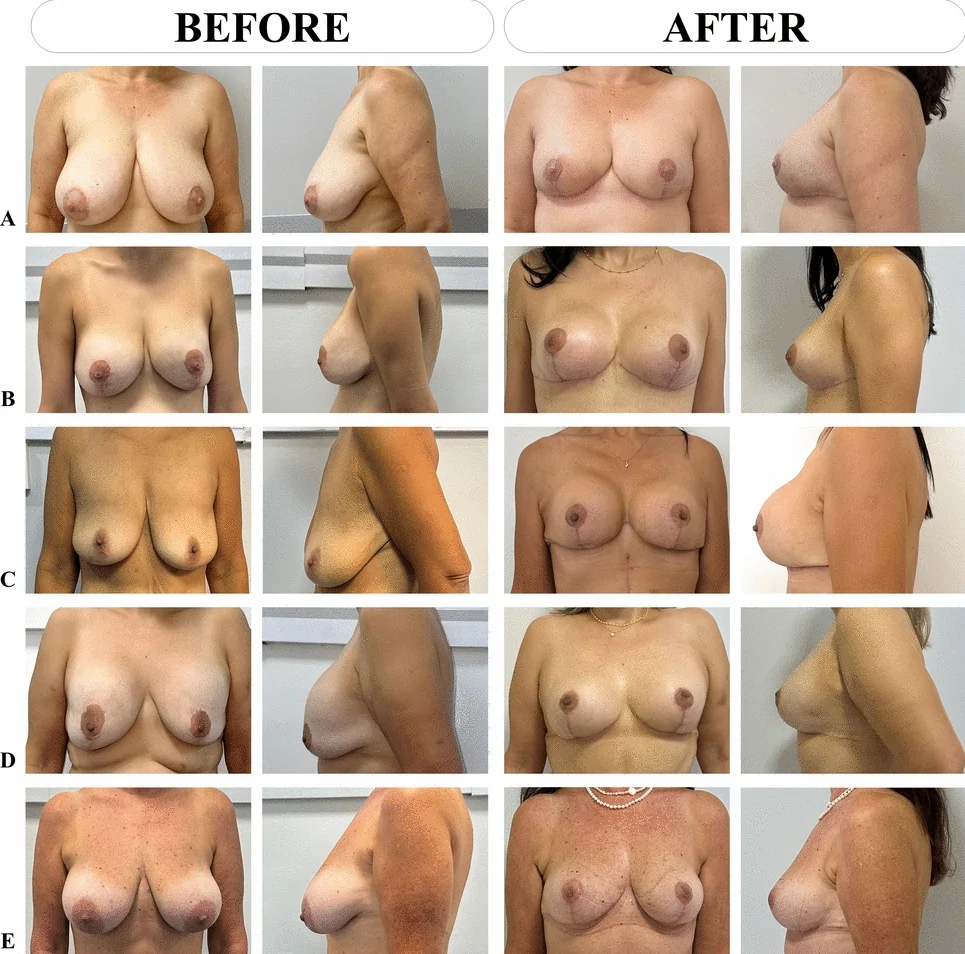
Preoperative and postoperative clinical photographs of patients undergoing aesthetic breast surgery with implant placement and reinforcement using the GalaFLEX® (poly-4-hydroxybutyrate) absorbable scaffold. Preoperative images (left columns) demonstrate common aesthetic concerns, including breast ptosis, inferior pole elongation, reduced upper pole fullness, and loss of anterior projection. Postoperative images (right columns) show repositioning of the breast mound with improved anterior projection, enhanced contour of the upper and lower poles, and better definition of the inframammary fold. Representative cases (**A–E**) illustrate improved breast symmetry and contour regularity following scaffold-assisted aesthetic breast surgery

Figure 3 presents preoperative and postoperative clinical photographs of patients submitted to aesthetic breast surgery with implant placement reinforced by an absorbable synthetic mesh. Preoperative images demonstrate typical aesthetic concerns, including breast ptosis of varying degrees, reduced upper pole fullness, inferior pole elongation, and loss of anterior projection. In several cases, lateral views reveal descent of the breast mound and limited definition of the inframammary fold, reflecting compromised soft-tissue support (Fig. 3A–E, left columns). Postoperative images obtained during follow-up show repositioning of the breast mound with improved anterior projection and enhanced upper and lower pole contour (Fig. 3A–E, right columns). Improved definition of the inframammary fold and more balanced breast proportions are evident in frontal views, while lateral views demonstrate maintenance of implant position and restoration of breast projection without inferior displacement. Across the presented cases, reinforcement of the inferior and lateral breast envelope contributed to improved contour regularity and symmetry in aesthetic breast surgery.

**Fig. 4:**
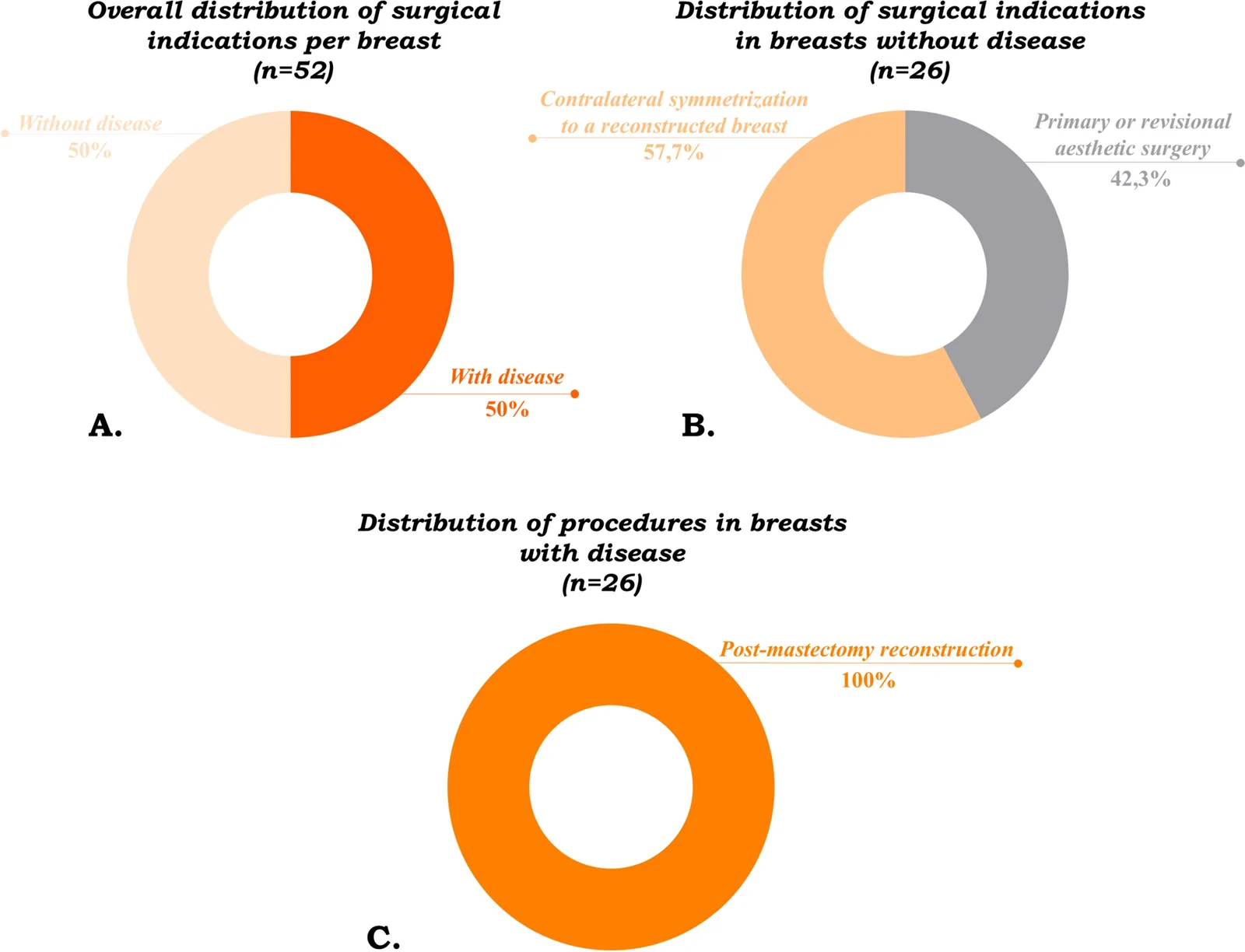
Distribution of surgical indications for mesh reinforcement according to breast disease status. Overall distribution of treated breasts (*n* = 52), demonstrating an equal proportion of breasts operated on in the presence and absence of disease (**A**). Indications among breasts without disease (*n* = 26) (**B**). In this subgroup, mesh reinforcement was primarily used for contralateral symmetrization in patients undergoing reconstruction on the opposite side, while the remaining cases corresponded to primary or revisional aesthetic breast surgery. No prophylactic procedures were recorded. Indications among breasts with disease (*n* = 26), showing that all cases were managed in the context of post-mastectomy breast reconstruction (**C**)

Regarding the clinical indications for scaffold use, treated breasts were evenly distributed between oncologic and non-oncologic cases. As shown in Fig. 4A, 50% of breasts were operated on in the presence of disease, whereas the remaining 50% corresponded to breasts without disease. Among breasts without disease, the primary indication for mesh reinforcement was contralateral symmetrization to a previously reconstructed breast, accounting for 57.7% of cases, while primary or revisional aesthetic procedures represented 42.3% (Fig. 4B). No prophylactic procedures were recorded in this subgroup. In contrast, all breasts classified as having disease were managed in the context of post-mastectomy reconstruction, representing 100% of procedures in this group (Fig. 4C).

**Fig. 5:**
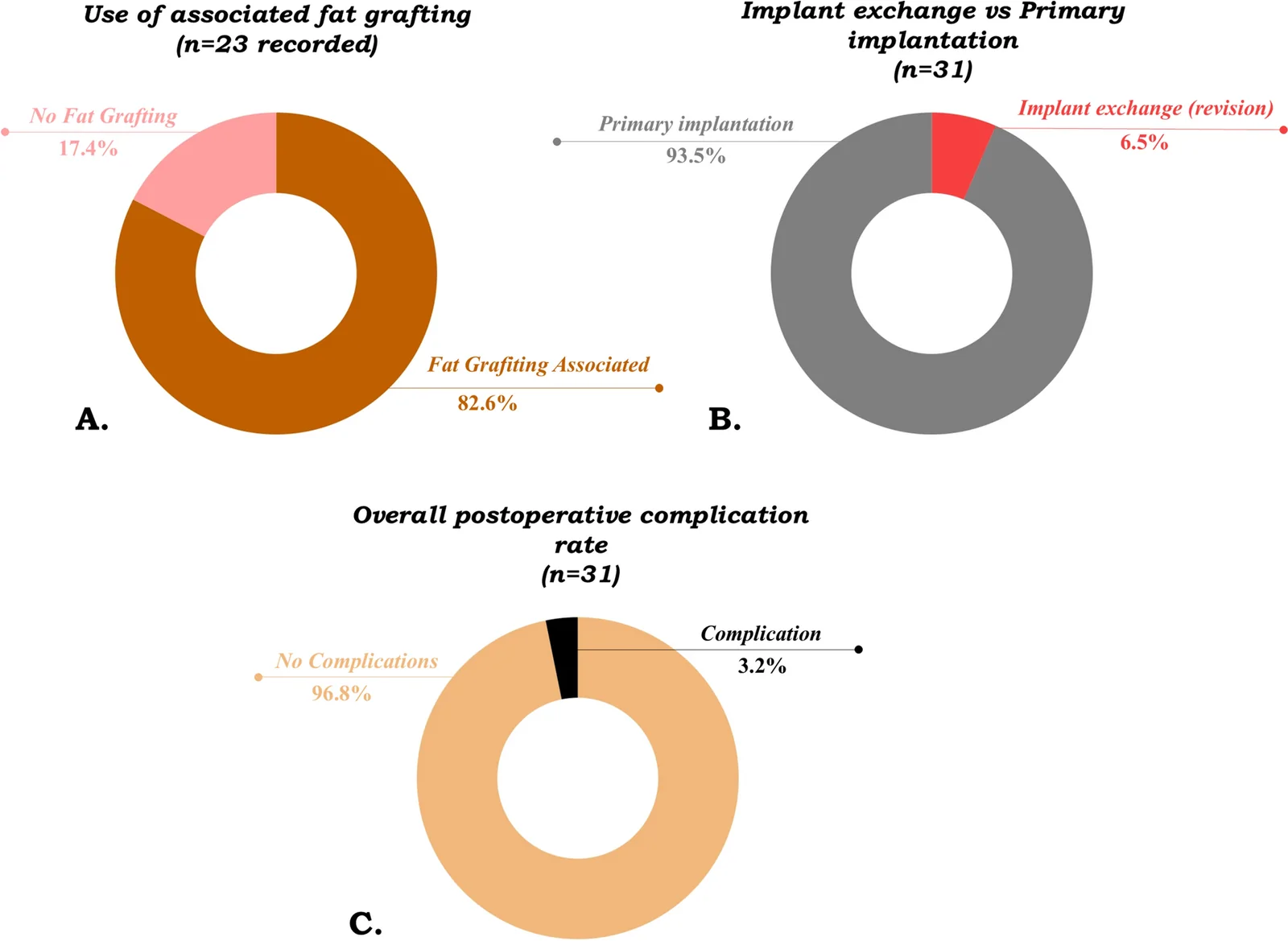
Distribution of adjunctive surgical techniques and overall postoperative outcomes following mesh reinforcement. Use of associated autologous fat grafting in the subset of procedures where this information was recorded (*n* = 23), showing that most breasts received additional fat grafting (**A**). Distribution of implant context (*n* = 31), comparing primary implant placement with implant exchange (revision surgery), demonstrating that the majority of procedures were performed as primary implantations (**B**). Overall postoperative complication rate in the cohort (*n* = 31), illustrating a low incidence of complications, with most patients experiencing an uneventful postoperative course (**C**)

Regarding surgical strategy and postoperative outcomes, adjunctive procedures and implant context varied across the cohort. As shown in Fig. 5A, autologous fat grafting was performed in the majority of cases in which this variable was recorded, accounting for 82.6% of procedures, while 17.4% of breasts did not receive associated fat grafting. Concerning implant context, most surgeries corresponded to primary implant placement. As illustrated in Fig. 5B, primary implantation represented 93.5% of procedures, whereas implant exchange (revision surgery) accounted for only 6.5% of cases. Postoperative safety outcomes are summarized in Fig. 5C. The overall complication rate was low, with 96.8% of patients experiencing no postoperative complications. A single complication was recorded, corresponding to 3.2% of the cohort.

## Discussion

The study demonstrated the clinical applicability of a bioresorbable P4HB mesh across a spectrum of breast surgical indications, with a substantial proportion of treated breasts undergoing reconstruction following oncologic surgery. In this cohort, half of the reinforced breasts corresponded to post-mastectomy reconstruction, underscoring that scaffold reinforcement was not restricted to aesthetic optimization but rather incorporated into cases in which soft-tissue support is most critical. This distribution reflects a broader shift in reconstructive practice, where biosynthetic materials are increasingly used to stabilize implant pockets and optimize lower pole support in oncologic scenarios [23]. The absence of a control group represents an important limitation of this study, precluding direct comparison with other reinforcement approaches, including permanent synthetic meshes or procedures performed without mesh support. The relatively small sample size and low event rate limit the ability to perform meaningful statistical analyses or risk stratification. In particular, subgroup analyses based on radiotherapy status, surgical indication (reconstructive versus aesthetic), or primary versus revision procedures were not performed. As a result, the findings should be interpreted as descriptive of early clinical experience rather than providing statistically robust conclusions. Larger studies with adequate power and predefined subgroup analyses will be necessary to better characterize outcomes across different clinical scenarios.

Breast reconstruction following cancer therapy presents unique challenges, once mastectomy flap thickness, prior or planned radiotherapy, and compromised tissue perfusion all contribute to an elevated risk of complications, including wound dehiscence, seroma, infection, capsular contracture, and implant loss [24, 25]. In this context, reinforcement materials must not only provide mechanical support but also integrate predictably without provoking chronic inflammation [26, 27]. The biosynthetic P4HB scaffold was developed to address this need by offering temporary structural reinforcement during the critical phases of healing, followed by gradual resorption and replacement with host collagenous tissue [10, 22]). Preclinical and clinical investigations have demonstrated that P4HB promotes constructive remodeling with progressive tissue ingrowth, while avoiding the long-term foreign body burden associated with permanent meshes [28–31].

Clinical literature evaluating P4HB meshes in breast surgery has expanded in recent years [28, 29]. Early reports described its use in aesthetic breast surgery for soft-tissue reinforcement and implant support, highlighting improved lower pole stability and reduced bottoming out in selected patients [32, 33]. Subsequent studies extended its application to reconstructive surgery, including tissue expander–based and direct-to-implant reconstructions, suggesting complication profiles comparable to ADM in selected cohorts [34–36]. Comparative analyses have reported similar rates of seroma, infection, and explanation between P4HB and biologic matrices, with the potential advantages of reduced cost and elimination of long-term implanted material [36, 37]. These findings support the concept that a resorbable scaffold can provide sufficient mechanical strength during the early postoperative period, when implant positioning and soft-tissue support are most critical [38]. Although all patients had a minimum follow-up of 6 months, this duration is insufficient to fully evaluate long-term outcomes, particularly given that P4HB undergoes gradual resorption over approximately 12–18 months. As a result, late complications such as implant malposition, bottoming-out, capsular contracture, and long-term structural stability, as well as potential improvements in soft-tissue coverage, may not yet be fully captured. Longer follow-up, ideally extending beyond the resorption period, will be necessary to determine whether early mechanical support translates into durable clinical outcomes. In addition, implant-related variables such as manufacturer, gel cohesivity, and complete distribution of implant characteristics were not consistently available. These factors may influence mechanical stability and complication rates and therefore represent an additional limitation of the present study.

The oncologic reconstruction setting is particularly relevant when considering scaffold behavior. Radiation therapy, which is common in breast cancer treatment, is associated with impaired wound healing and increased capsular contracture rates [39, 40]. The gradual resorption of P4HB may reduce long-term inflammatory stimuli compared with permanent synthetic meshes, while still providing early mechanical stability [30]. Although this study was not designed to directly evaluate outcomes stratified by radiation exposure, the consistent use of P4HB in post-mastectomy reconstruction within this cohort supports its feasibility in a higher-risk population. Importantly, all breasts classified as having disease in this series underwent reconstruction following oncologic treatment, reinforcing the scaffold’s role in this context rather than in purely aesthetic surgery.

Another notable aspect of contemporary breast reconstruction is the integration of adjunctive techniques such as autologous fat grafting. Fat grafting has been increasingly employed to improve contour, enhance flap thickness, and potentially mitigate radiation-related soft-tissue damage [41]. In this series, fat grafting was frequently combined with scaffold reinforcement, reflecting a multimodal approach in which structural support and volumetric refinement are used synergistically. This combined strategy has been described in other reports involving biosynthetic and biologic meshes and may contribute to improved pocket stability and long-term aesthetic outcomes [42, 43].

The innovation of P4HB lies in its balance between mechanical performance and biologic remodeling. Unlike ADMs, which rely on donor tissue processing and variable host integration, P4HB is a fully synthetic polymer produced through bioengineered processes, offering consistent material properties and avoiding risks related to biologic sourcing [34]. At the same time, its gradual resorption differentiates it from permanent meshes, which may remain as lifelong foreign bodies with potential long-term sequelae [34, 36, 37]. This intermediate profile, temporary support with eventual tissue replacement, is conceptually attractive in breast reconstruction, where long-term aesthetic outcomes depend on stable soft-tissue envelopes rather than permanent prosthetic reinforcement.

Despite these encouraging observations, some limitations must be acknowledged. This study represents a retrospective case series (Level of Evidence IV) without a control group, limiting the ability to draw definitive comparisons with ADM or other scaffold materials. Follow-up duration, while adequate for early postoperative assessment, may not capture late complications such as delayed capsular contracture or long-term implant malposition. Although all patients had a minimum follow-up of 6 months, this duration is insufficient to fully evaluate long-term outcomes, particularly given that P4HB undergoes gradual resorption over approximately 12–18 months. Late complications such as bottoming out, capsular contracture, or structural changes may therefore not yet be fully captured. Outcomes were not stratified according to radiotherapy status, despite a substantial proportion of patients having undergone preoperative radiotherapy. Given the well-established impact of radiation on wound healing and the risk of capsular contracture, this represents a limitation of the present study. The limited sample size and low event rate precluded meaningful subgroup analysis. Future studies with larger cohorts and appropriate stratification will be necessary to better evaluate the influence of radiotherapy on clinical outcomes.

Future research should aim to clarify the comparative effectiveness of P4HB scaffolds through prospective, multicenter studies with standardized reporting of patient risk factors, radiation exposure, and surgical variables. Longer-term follow-up will be essential to determine whether early benefits in pocket control and soft-tissue support translate into durable improvements in aesthetic and reconstructive outcomes. Comparative cost-effectiveness analyses may also help define the role of biosynthetic scaffolds relative to ADMs and other reinforcement strategies. Therefore, the findings of the present study should be interpreted as descriptive of early clinical experience rather than evidence of superiority over alternative materials or techniques.

## Conclusion

The use of bioresorbable scaffold reinforcement in breast surgery demonstrated a favorable early safety profile and broad applicability across different clinical scenarios. The material was predominantly used in oncologic breast reconstruction, while also being applied in aesthetic and symmetrization procedures in non-oncologic breasts. Adjunctive techniques, such as fat grafting, were frequently combined with mesh support, reflecting its integration into contemporary reconstructive strategies. Given the retrospective design, limited sample size, and short follow-up, these findings should be interpreted as descriptive of early clinical experience rather than evidence of clinical effectiveness. Further studies with longer follow-up and comparative designs are warranted to better define long-term outcomes and establish the role of scaffold-assisted breast surgery.
